# Higher purpose in life and education were associated with better cognition among older adults

**DOI:** 10.1055/s-0044-1779506

**Published:** 2024-02-23

**Authors:** Wellington Lourenço Oliveira, Ruth Caldeira de Melo, Meire Cachioni, Deusivania Vieira da Silva Falcão, Samila Sathler Tavares Batistoni, Tiago Nascimento Ordonez, Anita Liberalesso Neri, Mônica Sanches Yassuda

**Affiliations:** 1 Universidade de São Paulo, Escola de Artes, Ciências e Humanidades, Departamento de Gerontologia, São Paulo SP, Brazil.; 2 Universidade Estadual de Campinas, Faculdade de Ciências Médicas, Departamento de Gerontologia, Campinas SP, Brazil.

**Keywords:** Older Adults, Neuroticism, Depression, Purpose in Life, Cognition, Pessoas Idosas, Neuroticismo, Depressão, Propósito de Vida, Cognição

## Abstract

**Background** With aging, some cognitive abilities change because of neurobiological processes. Cognition may also be influenced by psychosocial aspects.

**Objective** To describe the relationship between a measure of neuroticism, depression symptoms, purpose in life, and cognitive performance in community-dwelling older adults.

**Methods** This was a cross-sectional analysis based on the data from the second wave of the Frailty in Brazilian Older Adults (FIBRA) study, carried out between 2016 and 2017. The sample consisted of 419 older people (≥ 72 years old) cognitively unimpaired and mostly with low education. The variables of interest were sociodemographic, Neuroticism domain from the NEO-PI-R, Geriatric Depression Scale (GDS), Purpose in Life (PiL) scale, and a cognitive composite score which included the Mini-Mental State Examination (MMSE), and the scores for the sub-items of the Mini-Addenbrooke's Cognitive Examination (M-ACE), namely, Verbal Fluency (VF) – Animal, Clock Drawing Test (CDT), Episodic Memory (name and address).

**Results** There was a greater number of women (70%), with older age (median = 80 years, IQR = 77-82), and low education (median = 4 years, IQR = 2-5). In the bivariate correlations, years of education (ρ = 0.415; p < 0.001) and PiL (ρ = 0.220; p < 0.001) were positively associated with cognition. Neuroticism (ρ = -0.175; p < 0.001) and depression symptoms (ρ = -0.185; p < 0.001) were negatively associated with cognition. In the logistic regression, after including confounding variables, the associations between cognition and PiL (OR = 2.04; p = 0.007) and education (OR = 1.32; p < 0.001) remained significant.

**Conclusion** Low PiL and low education levels were associated with worse cognition among older adults. Such results may be of relevance in programs that aim to improve cognition among older adults.

## Resumo

**Antecedentes** Com o envelhecimento, algumas habilidades cognitivas mudam devido a processos neurobiológicos. A cognição também pode ser influenciada por aspectos psicossociais.

**Objetivo** Descrever as relações entre uma medida de neuroticismo, sintomas depressivos, propósito de vida e o desempenho cognitivo em pessoas idosas residentes na comunidade.

**Métodos** Trata-se de uma análise transversal com base nos dados da segunda onda do estudo de Fragilidade em Idosos Brasileiros (FIBRA), realizado entre 2016 e 2017. A amostra foi composta por 419 pessoas idosas (≥ 72 anos) cognitivamente saudáveis e em maior parte com baixa escolaridade. As variáveis de interesse foram as sociodemográficas, domínio Neuroticismo do NEO-PI-R, Escala de Depressão Geriátrica (EDG) e Escala de Propósito de Vida (PV) e um escore cognitivo composto que incluiu o Miniexame de Estado Mental (MEEM) e as pontuações dos subitens do Miniexame Cognitivo de Addenbrooke (M-ACE), a saber, Fluência Verbal (FV) Animal, Teste do Desenho do Relógio (TDR) e Memória Episódica (nome e endereço).

**Resultados** Houve um maior número de mulheres (70%), com idade elevada (mediana = 80 anos, IIQ = 77-82) e baixa escolaridade (mediana = 4 anos, IIQ = 2-5). Nas correlações bivariadas, anos de escolaridade (ρ = 0,415; p < 0,001) e PV (ρ = 0,220; p < 0,001) foram positivamente associadas à cognição. Neuroticismo (ρ = -0,175; p < 0,001) e sintomas depressivos (ρ = -0,185; p < 0,001) foram negativamente associados à cognição. Na regressão logística, após a inclusão de variáveis de confusão, as associações entre cognição e PV (OR = 2,04; p = 0,007) e escolaridade (OR = 1,32; p < 0,001) permaneceram significativas.

**Conclusão** Baixo PV e baixa escolaridade foram associados à pior cognição em idosos. Tais resultados podem ser relevantes em programas que visam a melhorar a cognição entre pessoas idosas.

## INTRODUCTION

Age-related cognitive change is one of the most studied aspects of aging. With age, there may be a reduction in cognitive performance, with implications for daily life and well-being. 1 Cognitive changes are related to brain aging, that is, the anatomical and physiological changes that occur throughout the life cycle. 2 It has also been documented that cognitive performance in old age may be influenced by psychosocial aspects, such as personality profile, psychiatric conditions such as depression, and certain domains of psychological well-being - purpose in life (PiL), among other factors. 3 4 5

Among psychosocial aspects, studies have suggested that neuroticism, a personality factor related to negative emotional and behavioral reactions to stressful situations, has an association with cognitive performance. 6 This aspect of personality, although present in all individuals, when at elevated levels has often been linked to poor cognitive aging in longitudinal studies. For instance, Sutin et al. 7 reported that high scores in neuroticism among respondents were associated with worse performance in domains such as memory, function executive, attention, process speed, visuospatial ability, verbal fluency, and numerical reasoning. Another longitudinal study identified that low neuroticism was associated with less cognitive decline and greater longevity. 3

Depression is a mood disorder that significantly affects an individual's ability to function and relate to others. Depression symptoms (DS) have commonly been associated with reduced cognitive performance. DS can influence cognitive domains, such as learning and memory, attention and concentration, executive functions, and processing speed. 4 Also, in a longitudinal study, lifetime recurrent depression episodes were predictive of worse cognitive status, and a higher risk of cognitive impairment in early old age, 8 although this finding is contested by other studies (e.g., Burhanullah et al. 9 ).

PiL is one of the most important domains of the psychological well-being construct. It refers to the meaning, purpose, and sense of direction that people assign to their own lives. 10 Importantly, higher PiL is considered an important predictor of healthy aging. For instance, in previous work, Ryff and colleagues have documented that higher PiL predicts greater longevity and better health behaviors. 11 Recent research has shown that higher PiL can also have a positive impact on cognition. For example, in a longitudinal study, higher PiL was reported as having a protective effect against cognitive decline. 5 It was also associated with better coping skills during stressful events and greater longevity. 12 In a recent meta-analysis about PiL and cognition, PiL was associated with better performance in episodic memory and verbal fluency tasks. 13

The psychosocial aspects described above represent important mental health parameters that can significantly influence cognition among older people. 7 8 12 However, these data originated from high-income countries, where socioeconomic conditions and mental healthcare access tend to be significantly better than in middle- and low-income countries. Therefore, we understand that the association between psychosocial aspects and cognition should also be investigated in such adverse contexts. In addition, this association has rarely been explored among the oldest old, and no previous studies about the topic were identified in Brazil. Therefore, the objective of the present study was to investigate the associations between sociodemographic, neuroticism, DS, PiL variables, and cognitive performance in a sample of community-dwelling older adults, 72+ years, residing in the state of São Paulo, Brazil. We hypothesized that lower neuroticism, lower DS, and higher PiL would be associated with better cognitive performance.

## METHODS

### Participants and study design

The present study includes cross-sectional analyses of data from the second wave of the Frailty in Brazilian Older Adults (FIBRA) study, carried out between 2016 and 2017. The sample included older Brazilians aged 72+ years living in the city of Campinas and in the Ermelino Matarazzo district in the city of São Paulo, both located in the state of São Paulo.

The inclusion criteria for the follow-up participation were the same as for the baseline assessment:

• understanding instructions for tasks,
• accepting to participate in the study, and
• permanent residence in the census tract.

The exclusion criteria were as follows:

• impaired cognitive and communication skills, suggesting cognitive impairment;
• permanent or temporary inability to walk;
• aphasia or loss of muscle strength due to stroke sequelae;
• severe impairment of speech and motricity related to Parkinson's disease in an advanced stage;
• severe hearing and visual disability; and
• terminal disease. The informants were the older people themselves or a family member residing in the household. For more details regarding the FIBRA study please see Neri et al. 14

**Figure 1:**
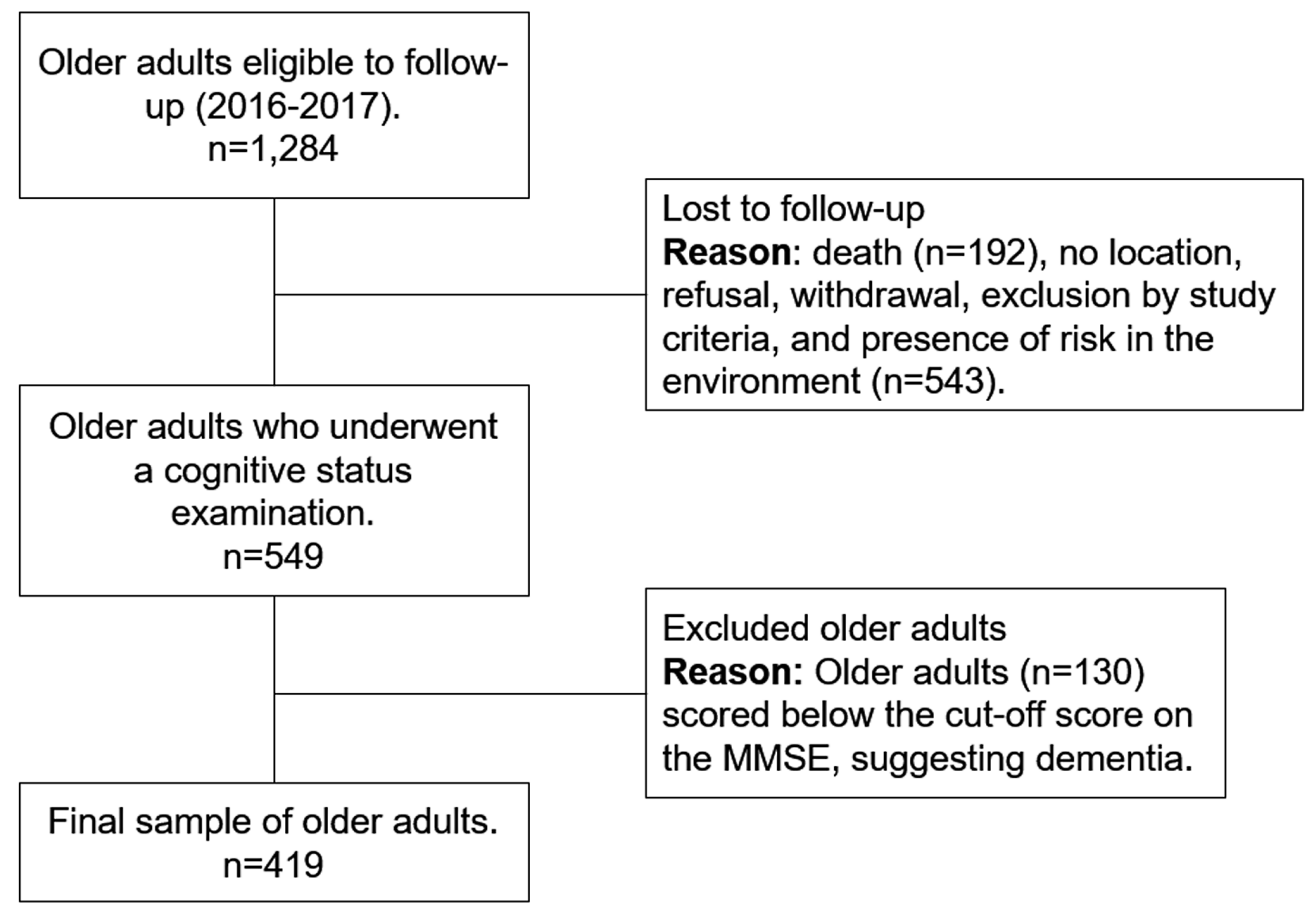
Recruitment flowchart and composition of the follow-up sample. FIBRA Study, 2016-2017.

Among the 1,284 participants at baseline, there were 192 deaths and 543 losses due to various reasons (participants not located [57.9%], refusals [3.5%], withdrawal of permission during data collection [5.5%], exclusion due to study criteria [1.6%], and presence of risks to interviewers [0.5%]). 14 A total of 549 older adults underwent assessment of cognitive status, of which 419 scored above the education-adjusted cut-off score for dementia on the Mini-Mental State Examination (MMSE) and completed the follow-up protocol 14 which included the measures of interest for the present study ( Figure 1 ). The adopted cutoff scores for the MMSE were: 17 points for illiterates, 22 points for 1–4 years of formal study, 24 points for 5–8 years, and 26 points for those with 9 or more. 15

The interviews were conducted by undergraduate and graduate students who were trained to apply the protocols. Recruitment was carried out in older adults' homes according to the list of addresses available in the baseline database. The evaluation was carried out at the residence by a pair of interviewers in one session, with an average duration of 75 minutes.

Participants or caregivers completed and signed an informed consent form. The project was approved by the ethical committee of the State University of Campinas, under the following protocol numbers - CAAE 49987615.3.0000.5404 and 92684517.5.1001.5404.

### Variables and instruments

#### *Sociodemographic data*

Age (72-79 and 80+ years old), sex, education (illiterate, 1-4 years of study, 5-8, and 9 + ), household living arrangement (live alone – yes or no), and family income were considered.

#### *MMSE*

This instrument assesses global cognition, with a maximum score of 30 points. 15

#### *Mini-Addenbrooke's Cognitive Examination (M-ACE)*

This is a brief cognitive screening instrument for dementia composed of temporal orientation questions, an Episodic Memory test (name and address), Verbal Fluency (VF) - animal category, and the Clock Drawing Test (CDT). 16

For the present analyses, the MMSE, and the M-ACE sub-item scores for VF, CDT, and the Episodic Memory delayed recall were used to compose a composite score for cognition. The M-ACE total score was not used to avoid the overlap of the temporal orientation sub-items present in the M-ACE and the MMSE.

#### *Neuroticism*

It represents the domain of the Revised NEO Personality Inventory (NEO-PI-R). It assesses the characteristics of emotional instability and individual adjustment. This scale consisted of 12 items rated on a Likert scale. The higher the score, the higher the intensity of neuroticism. 6 17 The FIBRA study did not collect data on other personality domains besides Neuroticism.

#### *Geriatric Depression Scale (GDS)*

This is a 15-item depression screening instrument for older adults. 18 A score ≥ 6 is considered to be suggestive of major depression.

#### *Purpose in Life (PiL)*

This scale is part of the Psychological Well-Being scale developed by Ryff and Keyes. 10 19 It examines the sense of meaning in life, beliefs that give meaning to life, and whether the person has goals for living. It consists of ten questions with responses on a Likert scale. The higher the score, the higher the purpose in life.

### Statistical analyses

The variables of interest in the study do not have a normal distribution, which is why non-parametric data was used. Therefore, for descriptive analysis of the data, the Mann-Whitey (two categories) and Kruskall-Wallis (three categories or more) tests were used. Mann-Whitney test and the Kruskall-Wallis (with post hoc comparisons, Dwass-Steel-Critchlow-Fligner [DSCF]) to compare continuous variables and the Chi-square test to compare categorical variables.

The dependent variable was the cognitive composite score including the MMSE and the VF, CDT, and Episodic Memory items from the M-ACE. To create the cognitive composite score, the individual test scores were normalized into z-scores. The z-scores for the tests were added and divided by the number of items, to generate a global cognitive score. Considering that the present sample was composed only of participants with unimpaired cognition, according to the MMSE, the composite score was transformed into a dichotomous variable, z ≤ 0 (newZ = 0) to represent lower cognitive performance and z > 0 (newZ = 1) to represent higher cognitive performance.

Spearman's correlation tests and logistic regression analyses were used to test the associations among the selected variables. For the logistic regression analysis, the cognitive composite score was the dependent variable, and the sociodemographic and psychosocial variables were included as independent variables. The forced entry model was used to include all the independent variables and the cognitive composite score in the regression equation.

The level of statistical significance adopted for all analyses was p < 0.05. The statistical software Jamovi (Version 2.3.28, 2022, Australia) and JASP (Version 0.17.2, 2023, The Netherlands) were used.

## RESULTS

The sample was comprised of cognitively unimpaired older adults, mostly with low education. Table 1 presents the sociodemographic, psychosocial, and cognitive characterization of the total sample and the sample divided by sex. Among the participants, 70% were women, with a high age (median = 80 years) and low educational level (median = 4 years), with 13% of participants without formal education. In addition, 18% of participants lived alone, with a greater number of women in this living condition. Also, 20% of participants had GDS scores suggestive of major depression and women had a higher level of neuroticism than men.

**Table 1:** Sociodemographic, psychosocial, and cognitive characterization of the total sample and divided by sex (n = 419). FIBRA Study, 2016-2017

Variables		Total n = 419 (100%)	Sex		p-value
			Women n = 293 (70%)	Men n = 126 (30%)	
**Age, n (%)**	MD (IQR)	80 (77-83)	80 (77-82)	80 (78-83)	0.064 ^+^
**Education, n (%)**	Illiterate	54 (13%)	42 (14%)	12 (10%)	0.194 ^++^
	Literate	356 (85%)	246 (84%)	110 (87%)	
	Missing	9 (2%)	5 (2%)	4 (3%)	
	MD (IQR)	4 (2-5)	4 (2-5)	4 (3-5.7)	0.221 ^+^
**Family income, n (%)**	MD (IQR)	2,400 (1,760-3,600)	2,325 (1,700-3,712)	2,500 (1,874-3,500)	0.876 ^+^
	Missing	47	33	14	
**Live alone, n (%)**	Yes	77 (18%)	61 (21%)	16 (13%)	0.013 ^++^
	No	239 (57%)	153 (52%)	86 (68%)	
	Missing	103 (25%)	79 (27%)	24 (19%)	
**MMSE**	MD (IQR)	25 (23-27)	25 (23-27)	26 (24-27)	0.108 ^+^
**VF**	MD (IQR)	11 (9-14)	11 (9-14)	11 (9-14)	0.872 ^+^
**CDT**	MD (IQR)	2 (1-4)	3 (1-4)	2 (1-4)	0.688 ^+^
	Missing	1	0	1	
**Episodic memory**	MD (IQR)	3 (0-5)	2 (0-5)	3 (1-5)	0.481 ^+^
	Missing	2	2	0	
**Neuroticism**	MD (IQR)	26 (21-31)	27 (22-32)	24 (19-29)	0.002 ^+^
	Missing	20	13	7	
**PiL**	MD (IQR)	3.6 (3.1-4)	3 (3.1-4)	3.5 (3.1-3.9)	0.467 ^+^
	Missing	17	11	6	
**GDS, n (%)**	≥6	82 (20%)	62 (21%)	20 (16%)	0.211 ^++^
	≤5	337 (80%)	231 (79%)	106 (84%)	
	MD (IQR)	3 (2-5)	3 (2-5)	3 (2-5)	0.407 ^+^

Abbreviations: CDT, Clock Drawing Test; GDS, Geriatric Depression Scale; IQR, interquartile range; MD, median; MMSE, Mini-Mental State Examination; n, sample size; PiL, Purpose in Life; VF, Verbal Fluence (animal category).

Note: ^+^ Mann Whitney test; ^++^ Qui-square test; p-value < 0.05 shows statistical significance.

Table 2 characterizes the sample divided by age groups. There were 185 individuals between 72 and 79 years of age (44%) and 234 older adults 80+ years. The younger group showed a lower score on Neuroticism (p = 0.050).

**Table 2:** Sociodemographic, psychosocial, and cognitive characterization of the total sample divided by age group (n = 419). FIBRA Study, 2016-2017

Variables		Age group		p-value
		72-79 n = 185 (44%)	80+ n = 234 (56%)	
**Education, n (%)**	Illiterate	24 (13%)	30 (13%)	0.931 ^++^
	Literate	156 (84%)	200 (85%)	
	Missing	5 (3%)	4 (2%)	
	MD (IQR)	4 (2-6)	4 (2-4)	0.159 ^+^
**Family income, n (%)**	MD (IQR)	2,346 (1,800-3,070)	2,490 (1,700-4,000)	0.628 ^+^
	Missing	17	30	
**Live alone, n (%)**	Yes	24 (13%)	53 (23%)	0.659 ^++^
	No	81 (44%)	158 (67%)	
	Missing	80 (43%)	23 (10%)	
**MMSE**	MD (IQR)	25 (23-27)	25 (23-27)	0.772 ^+^
**VF**	MD (IQR)	12 (9-14)	11 (8-13)	0.053 ^+^
**CDT**	MD (IQR)	2 (1-5)	3 (1-4)	0.242 ^+^
	Missing	0	1	
**Episodic memory**	MD (IQR)	2 (0-5)	3 (1-5)	0.817 ^+^
	Missing	0	2	
**Neuroticism**	MD (IQR)	27 (22-32)	25 (20-30)	0.050 ^+^
	Missing	7	13	
**PiL**	MD (IQR)	3.6 (3.1-4)	3.6 (3.1-4)	0.924 ^+^
	Missing	8	9	
**GDS, n (%)**	≥6	34 (18%)	48 (21%)	0.789 ^++^
	≤5	151 (82%)	186 (79%)	
	MD (IQR)	3 (2-5)	3 (2-5)	0.369 ^+^

Abbreviations: CDT, Clock Drawing Test; GDS, Geriatric Depression Scale; IQR, interquartile range; MD, median; MMSE, Mini-Mental State Examination; n, sample size; PiL, Purpose in Life; VF, Verbal Fluence (animal category).

Notes: ^+^ Mann Whitney test; ^++^ Qui-square test; p-value < 0.05 shows statistical significance.

Table 3 demonstrates the differences related to educational level (illiterate, 1-4 years of study, 5-8, and 9 + ). Among those with higher education (9+ years) income was higher and there was a higher percentage of people living alone. The higher the level of education of the participants, the higher the cognitive scores.

**Table 3:** Sociodemographic, psychosocial, and cognitive characterization of the total sample divided by educational level (n = 419). FIBRA Study, 2016-2017

Variables		Educational level				**p-value**
		Illiterate n = 57 (14%)	1-4 n = 244 (58%)	5-8 n = 68 (16%)	9+ n = 50 (12%)	
**Age, n (%)**	MD (IQR)	81 (76-84)	80 (77-82)	78 (75-81.3)	79.5 (77-83.8)	0.058*
**Family income, n (%)**	MD (IQR)	1,874 (1,264-2,811) ^c,d^	2,000 (1,625-3,000) ^d^	2,500 (1,800-4,000) ^d,a^	4,000 (2,950-7,000) ^b,c,a^	<0.001*
	Missing	12	26	6	3	
**Live alone, n (%)**	Yes	8 (14%)	41 (17%)	14 (21%)	14 (28%)	<0.001 ^++^
	No	36 (63%)	144 (59%)	39 (57%)	20 (40%)	
	Missing	13 (23%)	59 (24%)	15 (22%)	16 (32%)	
**MMSE**	MD (IQR)	20 (18-23) ^b,c,d^	25 (23-27) ^c,d,a^	26 (25-27.3) ^b,d,a^	27 (26-28) ^b,c,a^	<0.001*
**VF**	MD (IQR)	10 (8-12) ^c,d^	11 (9-14)	12 (9.7-14.3) ^a^	12 (11-16.8) ^a^	0.005*
**CDT**	MD (IQR)	0 (0-1) ^b,c,d^	3 (1-4) ^d,a^	3 (2-4) ^a^	4 (2-5) ^b,a^	<0.001*
	Missing	0	1	0	0	
**Episodic memory**	MD (IQR)	0 (0-3) ^b,c,d^	2 (1-5) ^d,a^	3 (1-5) ^a^	5 (3-6) ^b,a^	<0.001*
	Missing	1	1	0	0	
**Neuroticism**	MD (IQR)	27.5 (23.8-33.3)	26.5 (21-31)	24.5 (20-29)	25 (20-30)	0.229*
	Missing	1	12	6	1	
**PiL**	MD (IQR)	3.4 (3-3.9)	3.6 (3.1-4)	3.7 (3.2-4)	3.8 (3.2-4.3)	0.183*
	Missing	3	10	3	1	
**GDS, n (%)**	≥6	12 (21%)	49 (20%)	12 (18%)	9 (18%)	0.972 ^++^ 0.211*
	≤5	45 (79%)	195 (80%)	56 (82%)	41 (82%)	
	MD (IQR)	3 (2-5)	3 (2-5)	2 (1-4.2)	2 (1-4)	

Abbreviations: CDT, Clock Drawing Test; GDS, Geriatric Depression Scale; IQR, interquartile range; MD, median; MMSE, Mini-Mental State Examination; n, sample size; PiL, Purpose in Life; VF, Verbal Fluence (animal category).

Notes: *Kruskal-Wallis test (with post hoc comparisons, Dwass-Steel-Critchlow-Fligner [DSCF]); ^++^ Qui-square test; p-value < 0.05 shows statistical significance. a = different from illiterates (p < 0.05); b = different from 1-4 years (p < 0.05); c = different from 5-8 years (p < 0.05); d = different from 9+ years (p < 0.05).

Table 4 shows the Spearman ρ (rho) bivariate correlations between global cognition (composite score) and the psychosocial and sociodemographic variables. There was a significant correlation between global cognition and education, Neuroticism, PiL, and GDS scores. PiL was negatively correlated with GDS and Neuroticism. Still, Neuroticism was positively correlated with GDS scores; and global cognition and education were also positively correlated.

**Table 4:** Spearman's bivariate correlation between variables of interest in the sample (n = 419). FIBRA Study, 2016-2017

Variable 1		Variable 2	ρ	95% CI		p-value
				Lower	Upper	
Age	−	Education	-0.060	-0.155	0.036	0.217
Age	−	Family income	0.020	-0.082	0.121	0.704
Age	−	Global cognition	-0.068	-0.163	0.028	0.163
Age	−	Neuroticism	-0.057	-0.155	0.041	0.253
Age	−	PiL	-0.065	-0.162	0.033	0.195
Age	−	GDS	0.003	-0.092	0.099	0.945
Education	−	Family income	0.329	0.235	0.417	<0.001
Education	−	Global cognition	0.415	0.332	0.491	<0.001
Education	−	Neuroticism	-0.141	-0.236	-0.043	0.005
Education	−	PiL	0.077	-0.021	0.173	0.125
Education	−	GDS	-0.116	-0.210	-0.021	0.017
Family income	−	Global cognition	0.237	0.138	0.330	<0.001
Family income	−	Neuroticism	-0.148	-0.248	-0.045	0.005
Family income	−	PiL	0.128	0.025	0.228	0.015
Family income	−	GDS	-0.124	-0.223	-0.022	0.017
Global cognition	−	Neuroticism	-0.175	-0.268	-0.078	<0.001
Global cognition	−	PiL	0.220	0.125	0.311	<0.001
Global cognition	−	GDS	-0.185	-0.276	-0.091	<0.001
Neuroticism	−	PiL	-0.332	-0.418	-0.240	<0.001
Neuroticism	−	GDS	0.526	0.451	0.594	<0.001
PiL	−	GDS	-0.446	-0.521	-0.364	<0.001

Note. Global cognition (composite score = Mini-Mental State Examination + Verbal Fluence (animal category) + Clock Drawing Test + Episodic Memory (name and address)/4); PiL = Purpose in Life; GDS = Geriatric Depression Scale (total score); ρ = Spearman's rho; CI = confidence interval; p-value < 0.05 shows statistical significance.

Table 5 presents the results of the logistic regression analysis including the variables of interest (sociodemographic, psychosocial, and cognitive variables). This model did not show multilinearity (Tolerance > 0,1; VIF < 10) among the independent variables. The model indicated that education and PiL were significantly associated with global cognitive performance.

**Table 5:** Logistic regression including the study variables of interest with the cognitive composite score as the dependent variable (n = 419). FIBRA Study, 2016-2017

Parameter	Estimate	OR	95% CI		p-value
			Lower	Upper	
Age	-0.054	0.947	-0.115	0.006	0.079
Sex (men)	-0.186	0.831	-0.859	0.488	0.589
Education	0.279	1.321	0.168	0.389	<0.001
Family income	-0.000	1.000	-0.000	0.000	0.759
Live alone (yes)	0.243	1.275	-0.528	1.014	0.537
Neuroticism	-0.032	0.969	-0.075	0.011	0.148
GDS	0.078	1.081	-0.063	0.219	0.277
PiL	0.714	2.043	0.197	1.232	0.007

Note. GDS = Geriatric Depression Scale (total score); PiL = Purpose in Life; OR = odds ratio; CI = confidence interval; p-value < 0.05 shows statistical significance.

## DISCUSSION

This study explored the relationships between psychosocial and sociodemographic variables with cognitive performance in adults 72+ years. Among the main results, in bivariate correlations, global cognition was significantly related to education, Neuroticism, GDS, and PiL. In the logistic regression, with psychosocial and sociodemographic variables included as independent variables, global cognition was significantly associated with PiL and number of years of education.

In the sample, a greater presence of women and older people (80+ years) was observed, similarly to what was found in the study by Freitas et al., 20 among other studies, suggesting greater participation of women in gerontological research. The sample consisted mostly of participants with a low educational level. In Brazil, this has been a common finding in community-based studies, as it reflects the historical reality of lower access to education in older generations. 21 22

It was identified that 18% of participants lived alone, with a greater percentage of women in this living arrangement. In the National Health Survey in Brazil, carried out in 2013, it was observed that 15.3% of people aged 60 and over lived alone. 21 The change from a cohabitation profile towards a greater number of single-person households in recent decades is related to several factors, such as the demographic and epidemiological transition, changes in family arrangements, shorter duration of marital unions, and fewer children or more childless couples. 23

When the psychosocial variables were compared across sex, it was observed that women had a higher level of Neuroticism. This finding has been commonly reported, although, it is poorly understood, especially among older adults. 24

In the present study, participants with low education had worse cognitive performance, and global cognition and educational level were positively correlated, replicating the well-known negative impact of low education on cognition. 25 High education is understood as an important marker of cognitive reserve, that is, brain characteristics that allow higher cognitive functioning than expected in the face of pathological processes or aging. 26 Therefore, this finding was expected.

Elevated neuroticism has been associated with worse cognitive performance. For example, longitudinal analyses of the Health and Retirement Study (HRS) identified that among individuals with higher neuroticism, there was significantly lower performance in cognition. 7 Another study reported that high neuroticism was associated with lower scores on episodic memory. 27 In the present study, there was a significant negative correlation between global cognition and neuroticism. However, in the logistic regression, with the inclusion of other psychosocial and demographic variables, the association did not remain significant.

Similarly, cognition has been negatively associated with elevated DS in previous studies. 8 Several studies have suggested the negative influence of depression on cognitive performance. 28 29 Studies have shown deficits in memory and executive functions among people with major depression. 4 30 Studies have also pointed out that higher neuroticism and DS could be associated with faster cognitive decline with age 31 32 and a higher incidence of dementia. 33 However, Burhanullah et al. 9 found no association between depression and dementia. In the present analyses, GDS scores were negatively related to global cognition, yet this association was not significant in the multivariable model.

Differently from the other psychosocial variables in the present study, PiL was positively associated with global cognition in the bivariate correlation analyses and the association remained significant in the logistic regression, in the presence of other psychosocial and sociodemographic variables. This finding suggests that the influence of PiL on the cognition of older adults may be greater than the influence of DS and neuroticism. High PiL scores have previously been associated with better cognitive performance, in episodic memory and verbal fluency, and with a lower risk of cognitive decline. 34 35 It is possible that older people with higher levels of PiL have a more positive view of aging and present more protective behaviors in relation to their health. 36 In turn, such behaviors could be associated with greater cognitive reserve, such as involvement in intellectually stimulating activities.

Logistic regression results revealed that besides higher PiL, higher education was also significantly associated with better global cognition. These findings are in line with the Emory Healthy Aging Study, in which low PiL was identified as a robust predictor of cognitive decline, especially when associated with risk factors such as low education. 35

Additionally, it was possible to observe, in the bivariate correlations, that PiL was negatively correlated with GDS and Neuroticism. These findings were similar to those found in previous studies. In Ribeiro et al., 37 high PiL was associated with a lower number of SD and in Kim et al. 38 to a reduced risk for developing major depression. Other authors postulated the idea that higher PiL could lead to greater adoption of healthy behaviors, 39 a more positive view of aging and life, greater resilience, optimism, a sense of self-efficacy, and better functioning of biological systems. 36 Likewise, higher PiL would be related to low neuroticism, as it could enable emotional self-regulation in the management of adverse events 39 and also favor positive affects. 37

One of the limitations of this study is its cross-sectional design. Longitudinal surveys, with follow-up data over an extended period, could provide information on the trajectories of these variables and generate a greater understanding of such relationships. In addition, in the present study, cognition was investigated with the use of a composite score based on screening tests, which precluded the investigation of the association with specific cognitive domains. It is important to highlight that the validity and reliability of each measure included in the composite score were not assessed. Unfortunately, the FIBRA study did not include additional domains of the NEO-PI-R, such as conscientiousness, which would have been important to investigate. On the other hand, the present study fills an important gap, as the Brazilian gerontological literature lacks studies involving psychosocial variables and cognitive performance in cohorts of older adults. These data are relevant for cross-cultural comparisons.

In conclusion, the present study investigated the associations between psychosocial variables and cognitive performance among participants from the second wave of FIBRA study. Lower PiL and lower education levels were associated with worse cognition. The association between PiL and global cognitive scores is relevant as it suggests that interventions geared to increase PiL (e.g., Friedman et al. 40 ) might also have a positive effect on cognition.
